# Preparation and Photocatalytic Performance of MoS_2_/MoO_2_ Composite Catalyst

**DOI:** 10.3390/ma16114030

**Published:** 2023-05-28

**Authors:** Daoyu Dong, Weitao Yan, Yaqiu Tao, Yunfei Liu, Yinong Lu, Zhigang Pan

**Affiliations:** 1 College of Materials Science and Engineering, Nanjing Tech University, Nanjing 211800, China; 202061203301@njtech.edu.cn (D.D.); victor-yan@hotmail.com (W.Y.); taoyaqiu@njtech.edu.cn (Y.T.); yfliu@njtech.edu.cn (Y.L.); yinonglu@njtech.edu.cn (Y.L.); 2 State Key Laboratory of Materials-Oriented Chemical Engineering, Nanjing 211800, China

**Keywords:** MoS_2_, MoO_2_, photocatalysis, formic acid, hydrogen production

## Abstract

Solar energy is an inexhaustible clean energy providing a key solution to the dual challenges of energy and environmental crises. Graphite-like layered molybdenum disulfide (MoS_2_) is a promising photocatalytic material with three different crystal structures, 1T, 2H and 3R, each with distinct photoelectric properties. In this paper, 1T-MoS_2_ and 2H-MoS_2_, which are widely used in photocatalytic hydrogen evolution, were combined with MoO_2_ to form composite catalysts using a bottom-up one-step hydrothermal method. The microstructure and morphology of the composite catalysts were studied by XRD, SEM, BET, XPS and EIS. The prepared catalysts were used in the photocatalytic hydrogen evolution of formic acid. The results show that MoS_2_/MoO_2_ composite catalysts have an excellent catalytic effect on hydrogen evolution from formic acid. By analyzing the photocatalytic hydrogen production performance of composite catalysts, it suggests that the properties of MoS_2_ composite catalysts with different polymorphs are distinct, and different content of MoO_2_ also bring differences. Among the composite catalysts, 2H-MoS_2_/MoO_2_ composite catalysts with 48% MoO_2_ content show the best performance. The hydrogen yield is 960 µmol/h, which is 1.2 times pure 2H-MoS_2_ and two times pure MoO_2_. The hydrogen selectivity reaches 75%, which is 22% times higher than that of pure 2H-MoS_2_ and 30% higher than that of MoO_2_. The excellent performance of the 2H-MoS_2_/MoO_2_ composite catalyst is mainly due to the formation of the heterogeneous structure between MoS_2_ and MoO_2_, which improves the migration of photogenerated carriers and reduces the possibilities of recombination through the internal electric field. MoS_2_/MoO_2_ composite catalyst provides a cheap and efficient solution for photocatalytic hydrogen production from formic acid.

## 1. Introduction

With the progress of science and technology, while productivity has developed rapidly, problems such as environmental damage and energy shortage have also deteriorated. In order to relieve the dependence on fossil energy, cheap and clean energy is desirable. Many new energy technologies have emerged, but some of them will be severely limited by factors such as geography and policy. Among them, solar photocatalysis is a new promising sustainable technology [1,2]. Solar energy is an inexhaustible source of energy in nature. However, it has fluctuations in supply time and is not convenient to use. Therefore, we can convert solar energy into other forms that are easy to use and store—hydrogen energy. Small-molecule hydrogen storage materials, such as hydrazine hydrate [3,4], sodium borohydride [5,6], formic acid and other materials, have been extensively studied as hydrogen storage materials with high hydrogen content and stable properties. Formic acid, as a by-product of the organic chemical industry, can be prepared as a product in the catalytic reaction of carbon dioxide hydrogenation [7]. Besides having the advantages common to small molecule hydrogen storage materials, formic acid also has many distinct properties.

The commonly used catalysts for hydrogen production from formic acid are precious metals and composite catalysts with precious metal components [8,9]. Shaybanizadeh’s team [10] stabled Pd–Au bimetallic alloy nanoparticles stabilized on boron nitride nanosheets via the deposition–precipitation method. When the catalyst contains 3% Pd and 5% Au, respectively, a hydrogen selectivity of 100% was reached at 50 °C. However, high costs and complex preparation processes limit the use of catalysts containing precious metals.

The development of photocatalytic hydrogen production technology depends on the development of high-performance materials. Many semiconductor materials have been found to have the ability of water decomposition, such as TiO_2_ [11], ZnO [12,13], WO_3_ [14,15,16] and CdS [17]. MoS_2_ is a layered material consisting of alternating layers of Mo and S atoms [18,19]. The layers are connected by van der Waals forces, and the atoms within the layers are bonded by covalent bonds. The common MoS_2_ has three crystal structure types, namely the 1T phase of the trigonal system, the 3R phase of the rhombic system, and the 2H phase of the hexagonal system [20]. 1T-MoS_2_ exhibits strong metallic properties and has good electron transfer ability. However, 2H-MoS_2_ is a material with semiconductor properties, and its high carrier recombination rate leads to poor catalytic efficiency [21]. In addition, the 2H-MoS_2_ conduction band potential is greater than the reduction potential of H^+^/H_2_, which does not meet the requirements of photocatalytic HER [22].

Among Mo compounds, MoO_2_ with various valence states and HER activity stands out [23,24]. Oxygen vacancies with sufficient concentration generate a large number of free electrons, and the Fermi energy level of MoO_2_ is located in the Mo_4d_ orbital, resulting in a metallic property. Its excellent conductivity provides efficient carrier transfer [25]. If a material can be prepared to retain the advantages of MoS_2_ and MoO_2_, it will be a progressive step in the field of photocatalytic hydrogen evolution.

MoS_2_/MoO_2_ is considered a promising non-noble metal catalyst for hydrogen evolution reaction. By calcination, Zhang formed a Schottky heterojunction between MoS_2_ and MoO_2_, and the hydrogen yield of the composite product was 3.4 times that of pure MoS_2_ [26]. To further explore the role of MoO_2_ in the HER of MoS_2_, Wang used MoO_3_ with different morphologies as a molybdenum source to produce MoO_2_ and studied the effects of pH value, temperature and molybdenum-sulfur mole ratio on the morphology of the produced MoO_2_. The relevant electrochemical properties were tested in subsequent experiments. They believed that there were many active sites in S [27], which is the reason that HER catalytic performance is better. Kang et al. modified the MoS_2_/MoO_2_ ratio by soft annealing, which caused no change in the structure, yet an improvement in the hydrogen evolution efficiency [28].

In past research, researchers often prepared MoS_2_ and MoO_2_ separately and then composite the two. Such multi-step reaction often requires high temperature, which not only has high requirements of experimental equipment, but also complicated the operation steps, which is not conducive to repetition, and limits its further application in practice. The one-step hydrothermal synthesis has the advantages of simple operation, high repeatability, and low preparation cost.

In order to solve the above problems, the authors used ammonium molybdate tetrahydrate as molybdenum source and thiourea as sulfur source, and prepared 1T-MoS_2_ by bottom-up hydrothermal method. By increasing synthesis temperature and time, the phase transformation process of molybdenum sulfide from the 1T phase to the 2H phase was realized. On this basis, a certain amount of 5% diluted hydrochloric acid is introduced to adjust the environment suitable for MoO_2_ formation. The morphology, structure, surface area and valence state of the composite catalyst were analyzed by XRD, SEM, XPS and BET. Using formic acid as the hydrogen storage material, the photocatalytic performance of the composite catalysts was evaluated, and then the mechanism was explored. When 1T-MoS_2_ is combined with MoO_2_, the hydrogen selectivity improves to 72.5%, with the cost of a 10% decrease in hydrogen production. However, when 2H-MoS_2_ was combined with MoO_2_, hydrogen production and hydrogen selectivity were significantly improved. Though the hydrogen production rate and selectivity of the catalysts used in this work are much higher than those reported in the literature, the energy to irradiate the reaction was from the Unfiltered Hg lamp which contains a much higher amount of ultraviolet light. The catalysts studied in this work are applicable in high-concentration pollutant control in water source and hydrogen production under high irradiation.

## 2. Materials and Methods

### 2.1. Materials

Thiourea (CH_4_N_2_S) was purchased from Tianjin Zhiyuan Chemical Reagent Co. (Tianjin, China). Ammonium molybdate tetrahydrate ((NH_4_)_6_Mo_7_O_24_·4H_2_O, 99%) was purchased from Sinophosphate Chemical Reagent Co. (Tianjin, China). Hydrochloric acid (HCl) was supplied by Nanjing Chemical Specimen Co. (Nanjing, China). Formic acid (HCOOH) is supplied by Shanghai Lingfeng Chemical Reagent Co. (Shanghai, China). All chemicals are analytical grade and used without further purification.

### 2.2. Synthesis of Photocatalysts

MoS_2_ particles: 1T-MoS_2_ was synthesized by hydrothermal method. A total of 1.059 g of (NH_4_)_6_Mo_7_O_24_·4H_2_O and 2.284 g of CH_4_N_2_S(thiourea) were added in 60 mL deionized water at room temperature and was stirred until a clear solution was obtained. The solution was then transferred to a 100 mL Teflon-lined autoclave and held at 200 °C for 3 h. The resulting black precipitate was then dried at 40 °C for 8 h. For the preparation of 2H-MoS_2_, a similar procedure was applied except the hydrothermal temperature was increased to 240 °C and the duration was increased to 8 h.

MoO_2_ Flakes: 0.288 g of molybdenum powders and 0.384 g of molybdenum trioxide were added into a quartz boat and then calcined in a tubular furnace with an N_2_ atmosphere. The powder was heated to 700 °C at a rate of 5 °C/min and kept at 700 °C for 10 h. A black powder was obtained after cooling down to room temperature naturally.

MoS_2_/MoO_2_ composite catalyst: Since hydrothermal temperature is a crucial parameter to make the desired polymorph of MoS_2_, the same strategy was applied to prepare the MoS_2_/MoO_2_ composite catalyst with different polymorphs of MoS_2_. For the preparation of 1T-MoS_2_/MoO_2_, 1.0593 g of (NH_4_)_6_Mo_7_O_24_·4H_2_O and 0.4568 g of CH_4_N_2_S were dissolved in 40 mL deionized water at room temperature. After continuous stirring, an appropriate amount of 5% HCl was added to the solution. Then, the solution was moved into a 100 mL Teflon-lined autoclave and kept at 220 °C for 20 h. The HCl solution was used to control the amount of MoO_2_ produced and thus the MoS_2_/MoO_2_ ratio can be changed accordingly. Three 1T-MoS_2_/MoO_2_ samples with different ratios of MoS_2_/MoO_2_ were obtained using 18 mL, 20 mL and 22 mL of 5% HCl solution and the resulting products were denoted as 1T-18HCl, 1T-20HCl and 1T-22HCl, respectively. For the preparation of 2H-MoS_2_/MoO_2_ with different MoS_2_/MoO_2_ ratios 18 mL, 20 mL, 22 mL and 30 mL of 5% HCl solution was used, and the hydrothermal synthesis was carried out at 240 °C for 20 h. The resulting 2H-MoS_2_/MoO_2_ composites were named 2H-18HCl, 2H-20HCl, 2H-22HCl and 2H-30HCl.

### 2.3. Characterization

Powder X-ray diffraction patterns were obtained by Rigaku SmartLab diffractometer (Tokyo, Japan) using Cu Kα (λ = 0.154178 nm) as a radiation source. Rietveld refinement of XRD data was performed using GSAS-II (version 5511). The morphology of the samples was measured by scanning electron microscope (FESEM, Ultra-55, Zeiss, Oberkochen, Germany). Nitrogen adsorption-desorption isotherms were measured at −196 °C using a specific surface area and pore analyzer V-Sorb 1800 (NETZSCH-Gerätebau GmbH, Selb, Germany). The samples were pretreated at 105 °C for 12 h prior to measurement. The electrochemical impedance was measured at the electrochemical workstation CH1660E. X-ray photoelectron spectroscopy was recorded using Kratos AXIS Supra (Shimadzu, Kyoto, Japan).

### 2.4. Photocatalytic Degradation and Photoelectrochemical Test

The photocatalytic performance of the composite catalyst was evaluated by catalytic hydrogen evolution from formic acid under ultraviolet light using a 500 UV Hg lamp as the light source. For each test, 50 mg of the catalyst was dispersed in 500 mL of 10% formic acid solution. Prior to the test, the suspension was stirred in the dark for 30 min and the photocatalyst reactor was vacuumed to eliminate the air in the reactor. The produced gas was extracted every 5 min using a 500 µL gas sampler and the composition was analyzed using a gas chromatograph (SP-6890,Shandong Lunan Ruihong Chemical Instrument Co., Ltd., Tengzhou City, China). The amount of gas produced by photocatalysis was evaluated using the drainage method.

Electrochemical impedance measurement was conducted to study the electrochemical characteristics. A total of 20 mg of the catalyst was dispersed into 40 µL of a mixture of 5 wt% Nafion and 0.5 mL of anhydrous ethanol under ultrasound, and then 200 µL of the suspension was spread onto the surface of the conductive glass to prepare the working electrode. A total of 0.1 M Na_2_SO_4_ solution is used as the electrolyte, platinum electrode as the counter electrode and saturated glycerol electrode as the reference electrode Electrochemical impedance measurement was carried out using CH1660E electrochemical workstation.

## 3. Results and Discussion

### 3.1. Characterization and Properties of 1T-MoS_2_/MoO_2_ Composite Catalysts

#### 3.1.1. XRD Characterization

**Figure 1:**
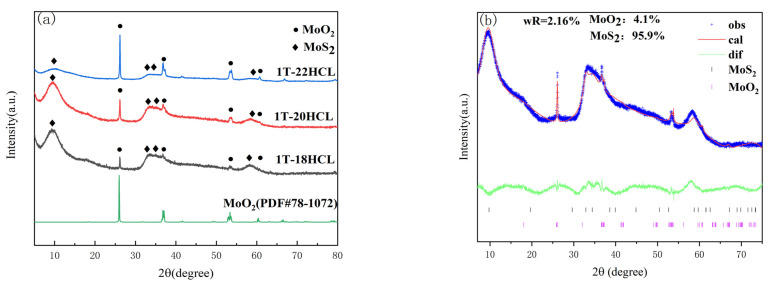

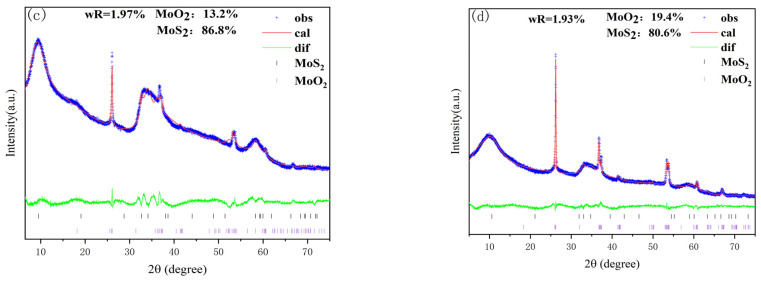
Powder X-ray diffraction pattern of 1T-MoS_2_/MoO_2_ composite catalyst, and the figure of fitting calculation; (**a**) Overlay of powder X-ray diffraction patterns of 1T composite catalyst (**b**) 1T-18; (**c**) 1T-20HCl; (**d**) 1T-22HCl.

Figure 1a shows the XRD patterns of 1T-MoS_2_/MoO_2_ composite catalyst with different MoO_2_ contents. It can be seen from Figure 1a that the characteristic peaks of the MoS_2_ component at 2θ equal to 9.6°, 33.1°, 35.4°, and 57.4° correspond to (001), (100), (102), and (110) crystallographic planes, respectively [29]. The characteristic peaks at 26.1°, 37.3°, 53.7°, and 61° correspond to (011), (211), (311), and (013) crystallographic planes of MoO_2_, respectively. In the composite sample of 1T-MoS_2_/MoO_2_, with the increase in the dosage of 5% HCl solution, the characteristic peak intensity of 1T-MoS_2_ at 2θ equal to 9.6° gradually decreases. On the contrary, the intensity of the characteristic peaks of MoO_2_ at 2θ equal to 26.1° and 37.7° gradually increases.

Rietveld quantitative analysis was carried out using the GSAS-II software package (version 5511) [30,31]. Figure 1b–d are the Rietveld plots of 1T-MoS_2_/MoO_2_ with different MoO_2_ contents. The blue line represents the raw XRD data obtained from the actual test, the red line is the data calculated by the software, the green line is the difference between the actual data and the fitted data, and the different colored tick marks represent the Bragg peak positions of different substances. The R_wp_ after refinement are 2.16%, 1.97%, and 1.93% for 1T-18HCl, 1T-20HCl and 1T-22HCl, respectively, indicating a good fit was obtained. The contents for each substance in 1T-MoS_2_/MoO_2_ are listed in Table 1. Rietveld quantitative analysis suggests that the content of MoO_2_ is positively correlated with the amount of dilute hydrochloric acid used.

#### 3.1.2. Morphology Analysis

**Figure 2:**
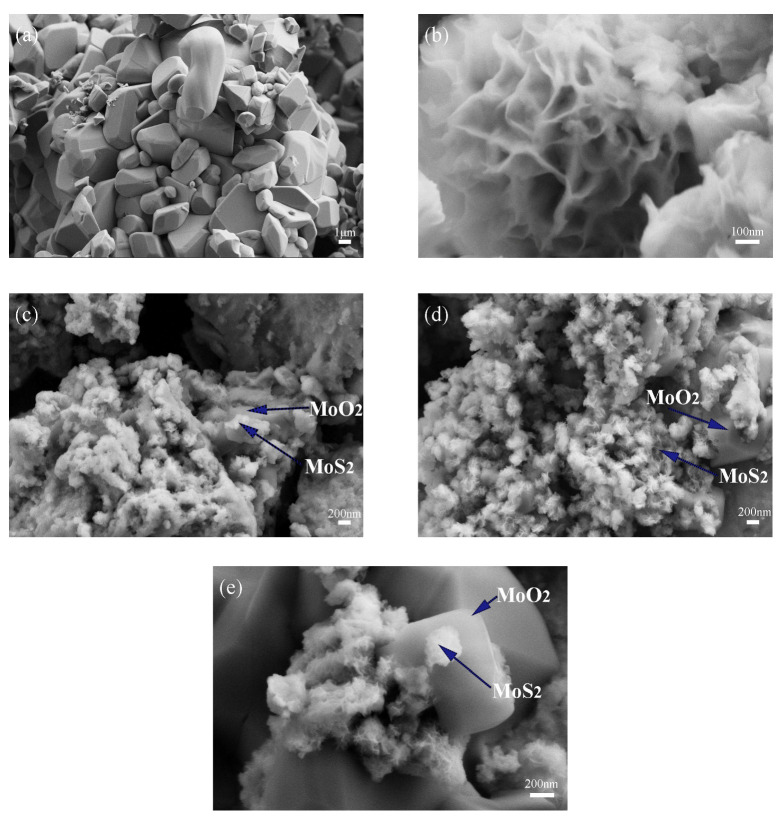
SEM images of (**a**) MoO_2_; (**b**) 1T-MoS_2_; (**c**) 1T-18HCl; (**d**) 1T-20HCl; (**e**) 1T-22HCl.

Different additions of 5% HCl solution will cause changes in the composition of the composite catalyst as described in the preceding section. Further characterization of morphology is carried out and Figure 2 shows scanning electron microscope images of three 1T-MoS_2_/MoO_2_ composite catalysts together with the images of 1T-MoS_2_ and MoO_2_ for comparison. As shown in Figure 2a, MoO_2_ exhibits a regular shape with a size of about 1μm, while 1T-MoS_2_ presents a nano-flower-like morphology with abundant petals shown in Figure 2b. Figure 2c shows the SEM image of 1T-18HCl, which is basically dominated by 1T-MoS_2_ nanoflowers. In Figure 2d, more MoO_2_ can be seen, while nano-flower-like MoS_2_ grows on block MoO_2_. In Figure 2e, the flower-like 1T-MoS_2_ still grows on the block MoO_2_, but more block MoO_2_ can be seen. It can be seen from Figure 2 that HCl promotes the growth of MoO_2_ in the composite catalyst. The SEM study is consistent with the results of the Rietveld quantitative analysis using GSAS-II.

#### 3.1.3. Electrochemical Impedance Measurement

**Figure 3:**
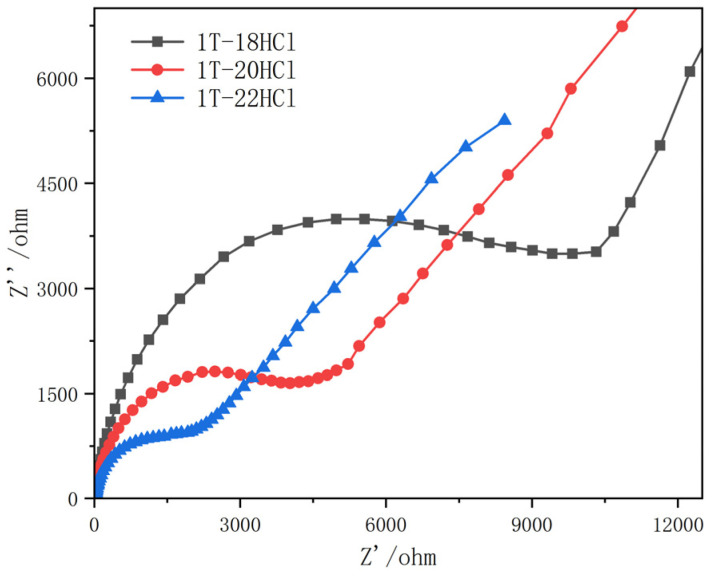
Electrochemical impedance of 1TMoS_2_/MoO_2_ composite catalyst with different MoO_2_ contents.

Figure 3 shows the electrochemical impedance measurements of the 1T-18HCl, 1T-20HCl, and 1T-22HCl composite catalysts. The arc radius of the high-frequency region of the measured electrochemical impedance represents the photoexcited carrier transfer efficiency of the material. The smaller the curvature radius, the lower the electrical impedance of the catalyst and the higher the transfer efficiency of the electron–hole pair. The results show that the impedance radius of 1T-18HCl is large, indicating that the transfer efficiency of electron–hole pairs is low. The radius of 1T-20HCl is smaller than that of 1T-18HCl, while the radius of 1T-22HCl is significantly smaller than that of 1T-20HCl and 1T-18HCl. The electrochemical impedance measurement showed that the electron transfer efficiency of the composite catalyst increased with the increase in MoO_2_ content.

#### 3.1.4. Photocatalytic Performance

**Figure 4:**
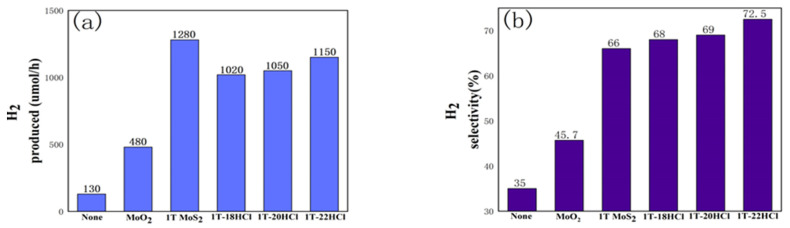
Histogram of photocatalytic performance comparison of different catalysts, (**a**) hydrogen production comparison chart, (**b**) hydrogen selectivity comparison chart.

The photocatalytic performance of hydrogen evolution from formic acid using 1T-MoS_2_/MoO_2_ composite catalysts is summarized in Figure 4. It can be seen from Figure 4 that in the composite catalyst, the hydrogen production and hydrogen selectivity will increase with the increase in MoO_2_ content. 1T-22HCl shows the best hydrogen selectivity (73%) which is increased by 7% and 27% compared with that of pure 1T-MoS_2_ and MoO_2_, respectively. However, in terms of hydrogen production per unit time, the composite catalyst is inferior to pure 1T-MoS_2_.

### 3.2. Characterization and Properties of 2H-MoS_2_/MoO_2_

#### 3.2.1. XRD Characterization

**Figure 5:**
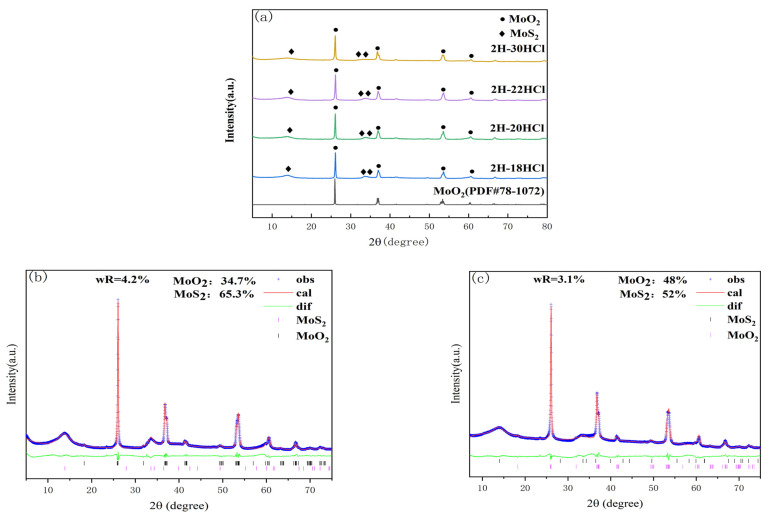
Powder X-ray diffraction pattern of 2H-MoS_2_/MoO_2_ composite catalyst, and the figure of fitting calculation; (**a**) Overlay of powder X-ray diffraction patterns of 2H composite catalyst (**b**) 2H-20HCl; (**c**) 2H-30HCl.

Figure 5a shows the XRD patterns of the 2H-MoS_2_/MoO_2_ composite catalysts prepared at 240 °C for 20 h, and the addition of 5% dilute HCl was 18, 20, 24, and 30 mL. It can be seen from Figure 5a shows the XRD patterns of 2H-MoS_2_/MoO_2_ composite catalysts with different MoO_2_ contents. It can be seen from Figure 5a that the characteristic peaks of the MoS_2_ component at 2θ equal to14°, 32.3°, 35.4°, and 57.4° correspond to (002), (100), (103), and (110) crystallographic planes, respectively. The characteristic peaks at 26.1°, 37.3°, 53.7°, and 61° correspond to (011), (211), (311), and (013) crystallographic planes of MoO_2_, respectively.

Rietveld quantitative analysis was carried out between the differentiated 2H-30HCl and the intermediate value of 2H-20HCl. The blue line represents the raw XRD data obtained from the actual test, the red line is the data calculated by the software, the green line is the difference between the actual data and the fitted data, and the different colored tick marks represent the Bragg peak positions of different substances. The smaller R_wp_ after refining were 4.2% and 3.1%, respectively, indicating a good fit. Table 2 lists the content of each substance in 2H-MoS_2_/MoO_2_. Rietveld quantitative analysis showed that the content of MoO_2_ was positively correlated with the amount of dilute chloric acid.

The R_wp_ of Rietveld quantitative analysis for 2H-20HCl and 2H-30HCl are 4.2% and 3.1%, respectively, indicating that the fittings are reliable. The results of the analysis illustrate that the content of MoO_2_ in 2H-30HCl is significantly more than that in 2H-20HCl. This agrees well with the fact that HCl can promote the formation of MoO_2_ as indicated by the preparation for 1T-MoS_2_/MoO_2_.

#### 3.2.2. Morphology Analysis of 2H-MoS_2_/MoO_2_

**Figure 6:**
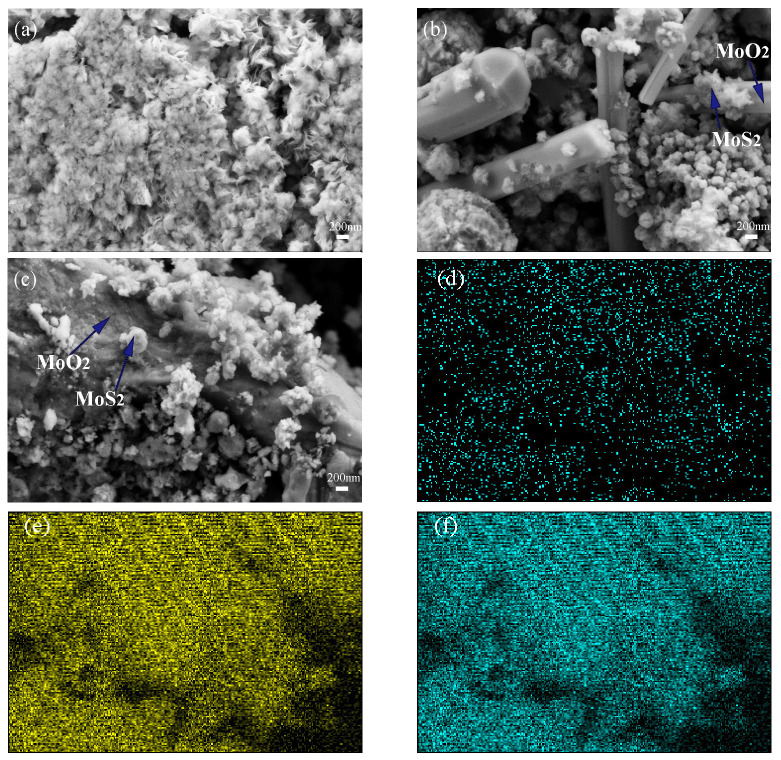
Scanning electron microscopy of 2H-MoS_2_, composite catalyst, and energy spectrum of 2H-30HCl (**a**) 2H-MoS_2_ (**b**) 2H-20HCl (**c**) 2H-30HCl (**d**) oxygen element (**e**) sulfur element (**f**) molybdenum element.

The scanning electron microscopy images of 2H-20HCl and 2H-30HCl are shown in Figure 6. Due to a longer period of high temperature in hydrothermal treatment, it is difficult for MoS_2_ to maintain the flower-like morphology, and the agglomeration of crystal grains is intensified. Instead, MoS_2_ nanospheres are observed. Figure 6b shows that MoO_2_ transforms into a rod-like morphology when the temperature is 240 °C. Figure 6c shows a similar rod-like shape for MoO_2_ in 2H-30HCl compared with that of 2H-20HCl, indicating excess HCl have little effect on the morphology of MoO_2_. 2H-20HCl and 2H-30HCl exhibit similar morphology for MoS_2_.

Figure 6d,e is the EDS elemental mapping of 2H-30HCl, it can be seen that S, Mo, and O elements are evenly distributed on the surface of the catalyst material. The elemental analysis using EDS is listed in Table 3, which is approximately consistent with the quantitative analysis using the Rietveld method.

#### 3.2.3. BET Measurements

Table 4 shows the specific surface area results obtained by the catalyst after nitrogen adsorption-desorption, and it can be seen from the data in Table 4 that the specific surface area of 2H-20HCl is 10% larger than that of 2H-30HCl. According to the calculated average pore size, both 2H-20HCl and 2H-30HCl belong to mesoporous materials.

#### 3.2.4. Electrochemical Impedance Measurement

**Figure 7:**
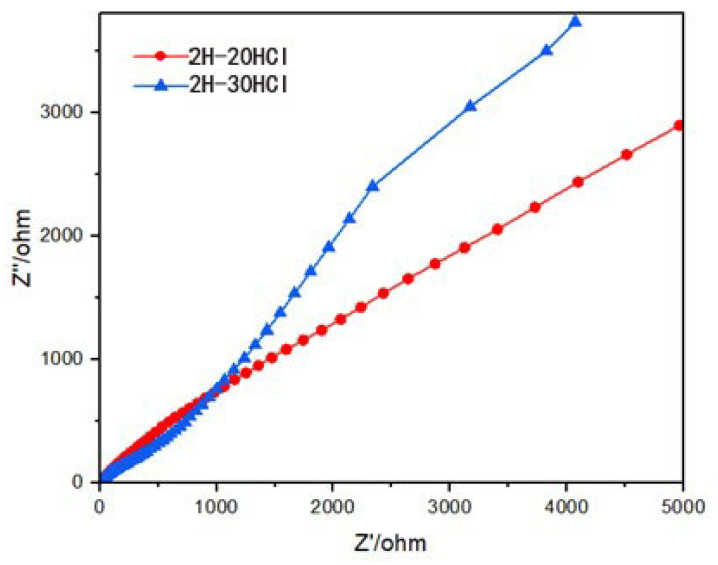
Electrochemical impedance diagrams of 2H-MoS_2_/MoO_2_ composite catalysts with different MoO_2_ contents.

The electrochemical impedance measured results of the 2H-MoS_2_/MoO_2_ composite catalyst are shown in Figure 7. Compared with 2H-30HCl, the curve of 2H-20HCl is closer to a straight line, indicating that its radius of curvature is greater than that of 2H-30HCl, that is, the charge transfer resistance is greater than 2H-30HCl, and the charge transfer resistance and the photocatalytic performance of the material often show a negative correlation. It is speculated that the photocatalytic performance of 2H-20HCl is not as good as that of 2H-30HCl. In summary, the decrease in pH caused by the addition of dilute hydrochloric acid can increase the MoO_2_ content in the 2H composite catalyst, and with the increase in MoO_2_ content, the charge transfer resistance of the material decreases.

#### 3.2.5. X-ray Photoelectronic Spectrum Analysis of 2H-MoS_2_/MoO_2_

**Figure 8:**
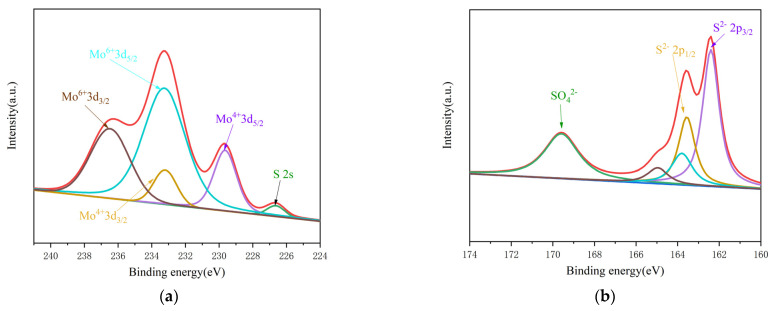
XPS diagram of catalyst 2H−30HCl (**a**) Peak fitting for Mo 3d; (**b**) Peak fitting for S 2P.

XPS analysis was performed for 2H-20HCl and 2H-30HCl. The spectrum for each sample shows similar characteristics and 2H-30HCl is discussed as an example. Figure 8 shows a narrow spectrum. (Please view the Figure S1 for the full spectrum) The peak fitting of Mo 3d and S 2p are shown in Figure 8a,b. As to the S 2p spec, the characteristic doublets at 162.4 eV, 163.6 eV,163.8 eV and 166.0 eV correspond to S^2−^ [32], while the band at 169.6 eV indicates that S is partially oxidized [33]. Figure 8a, the band corresponding to 233.3 eV is formed by Mo^6+^ spin orbit, which can be attributed to the slight oxidation of the sample surface [34,35]. The band at other positions such as 229.6 eV and 233.2 eV conform to the characteristic peak regularity of Mo^4+^3d_5/2_ and Mo^4+^3d_3/2_, and a characteristic band belonging to Mo^6+^3d_3/2_ appears near 236.5 eV. The small band at 226.7 eV is the interference peak of S 2 s, which indicates that MoS_2_ exists on the surface of the detected substance. Calculated by the XPS data, the molar ratio of Mo: S is 1:1.50, larger than the stoichiometric ratio of pure MoS_2_ (1:2), providing direct evidence for the incomplete sulfurization of MoO_2_.

#### 3.2.6. Photocatalytic Performance

**Figure 9:**
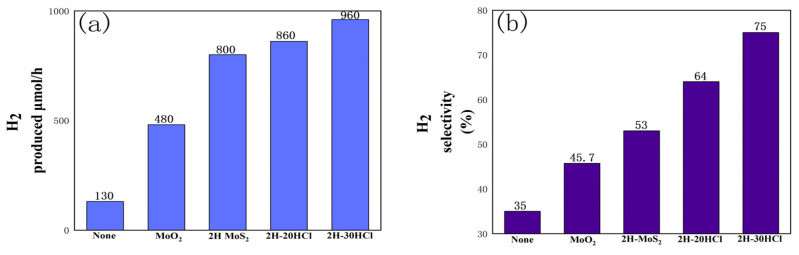
Histogram of photocatalytic performance comparison of 2H-MoS_2_/MoO_2_ catalysts with different MoO_2_ content, (**a**) comparison of hydrogen production;(**b**) comparison of hydrogen selectivity.

Figure 9 is a performance of the photocatalytic hydrogen production from formic acid using a 2H-MoS_2_/MoO_2_ composite catalyst. When the ratio of MoO_2_ to 2H-MoS_2_ in the sample changes, the selectivity of hydrogen increases. For 2H-30HCl, hydrogen selectivity reaches 75%, which is 2.14 times that of catalyst-free conditions and 1.42 times that of pure 2H-MoS_2_. The hydrogen production per unit time of the 2H composite catalyst is also higher than that of pure 2H-MoS_2_. Taking 2H-30HCl as an example, its hydrogen production has reached 960µmol/h, which is not only twice that of pure MoO_2_, but also higher than that of pure 2H-MoS_2_.

## 4. Conclusions

MoS_2_ of different polymorphs can be synthesized under appropriate hydrothermal conditions. 1T-MoS_2_ can be obtained at 200 °C for 3 h while 2H-MoS_2_ is formed at 240 °C for 8 h. MoS_2_/MoO_2_ composite catalysts can be synthesized by a one-step hydrothermal method. The polymorph of MoS_2_ in the composite can be adjusted using a similar strategy for the synthesis of pure 1T-MoS_2_ and 2H-MoS_2_.

Hydrochloric acid promotes the formation of MoO_2_ in the process of preparing MoS_2_/MoO_2_ composite catalysts. In the process of composite catalyst generation, an acidic environment promotes the formation of MoO_3_ from ammonium molybdate in aqueous solution. MoO_3_ is then reduced by thiourea to form MoO_2_, and then MoO_2_ is further vulcanized to produce MoS_2_. When the amount of hydrochloric acid is low, the MoO_x_ produced will be completely vulcanized to MoS_2_. With the gradual increase in the dosage of hydrochloric acid, the thiourea involved in sulfide MoO_x_ was gradually reduced, and the content of MoO_2_ phase in the product was gradually increased. Therefore, the ratio of MoS_2_ to MoO_2_ in the composite catalysts can be modified using different amounts of hydrochloric acid.

Comparing 1T-MoS_2_/MoO_2_ and 2H-MoS_2_/MoO_2_, it can be found that each has its own advantages. Based on the relatively high hydrogen production of 1T-MoS_2_, the overall hydrogen production per unit time of 1T-MoS_2_/MoO_2_ is higher than that of 2H-MoS_2_/MoO_2_, and hydrogen production on 1T-22HCl reached 1150 µmol/h. The advantage of 2H-MoS_2_/MoO_2_ is presented in its hydrogen selectivity, with a hydrogen selectivity of 75% for 2H-30HCl.

Due to the synergistic effect, the hydrogen selectivity of composite catalyst 1T-MoS_2_/MoO_2_ is improved compared to pure 1T-MoS_2_ and MoO_2_. The hydrogen selectivity also increases with the increase in MoO_2_ content, though the hydrogen yield on 1T-MoS_2_/MoO_2_ is lower than that of pure 1T-MoS_2_, which may be caused by the reduction in active sites from 1T-MoS_2_ with the addition of MoO_2_ [27]. However, with the increase in the amount of composite MoO_2_, the synergistic effect increases, and the hydrogen yield of the composite catalyst increases gradually.

P-type semiconductor 2H-MoS_2_ has a similar crystal structure matching lattice constant and crossed band gaps to N-type semiconductor MoO_2_. A heterostructure of 2H-MoS_2_ and MoO_2_ is formed [36,37]. Due to the formation of heterojunctions, oxidation and reduction occur in different components of the composite catalyst, which greatly reduced the recombination rate of charge carriers. Moreover, due to the presence of a junction electric field, the migration efficiency of charge carriers has also been significantly improved. Therefore, the photocatalytic performance of 2H-MoS_2_/MoO_2_ is better than that of pure 2H-MoS_2_ and MoO_2_.

## Figures and Tables

**Table 1:** The content of different substances in 1T-MoS_2_/MoO_2_ in different amounts of hydrochloric acid.

Sample	1T-MoS_2_/%	MoO_2_/%
1T-18HCl	95.9	4.1
1T-20HCl	86.8	13.2
1T-22HCl	80.6	19.4

**Table 2:** The content of different substances in 2H-MoS_2_/MoO_2_ in different amounts of hydrochloric acid.

Sample	2H-MoS_2_/%	MoO_2_/%
2H-20HCl	65.3	34.7
2H-30HCl	52	48

**Table 3:** Elemental content for EDS analysis of 2H-30HCl.

Element	Element wt%
O	28.18
S	41.82
Mo	30

**Table 4:** BET analysis of composite catalysts with different dilute hydrochloric acid additions.

Catalysts	BET Surface Area (m^2^/g)	Average Pore Width (nm)	Total Pore Volume (cm^3^/g)
2H-20HCl	6.632	24.34	0.040
2H-30HCl	6.019	14.97	0.023

## Data Availability

The data presented in this study are available on request from the corresponding author.

## Supplementary Materials

The following supporting information can be downloaded at: https://www.mdpi.com/article/10.3390/ma16114030/s1, Figure S1: Full-spectrum scanning of the XPS.
