# Supplementary material for: Preparation and Photocatalytic Performance of MoS2/MoO2 Composite Catalyst
Source: Materials (Basel). 2023 May 28;16(11):4030. doi: 10.3390/ma16114030 (PMC10254472; doi:10.3390/ma16114030)
Supplement: Supplementary file 1 [file materials-16-04030-s001.zip › materials-2397845-supplementary.pdf]

materials

Supplementary Materials

Preparation and photocatalytic performance of MoS_2/MoO_2 composite catalyst

Daoyu Dong [1], Weitao Yan [1,2], Yaqiu Tao [1,2], Yunfei Liu [1,2], Yinong Lu [1,2] and Zhigang Pan [1,2,*]

[1] College of Materials Science and Engineering, Nanjing Tech University, Nanjing 211800, China; 202061203301@njtech.edu.cn (D.D); victor-yan@hotmail.com(W.Y);taoyaqiu@njtech.edu.cn (Y.T.); yfliu@njtech.edu.cn (Y.L); yinonglu@njtech.edu.cn (Y.L.); panzhigang@njtech.edu.cn (Z.P.)
[2] State Key Laboratory of Materials-Oriented Chemical Engineering, Nanjing 211800, China
* Correspondence: panzhigang@njtech.edu.cn; Tel.: +86-138-5181-9674

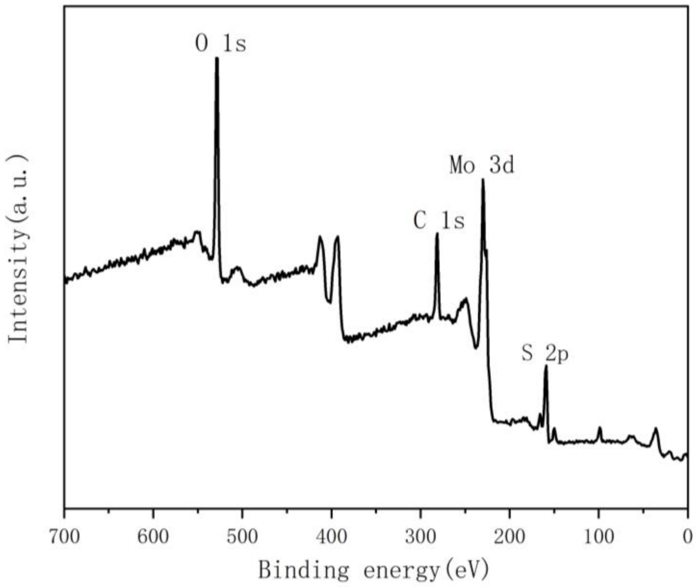

Figure S1. Full-spectrum scanning of the XPS.
